# 脑脊液ctDNA对非小细胞肺癌脑膜转移患者的临床价值

**DOI:** 10.3779/j.issn.1009-3419.2020.102.42

**Published:** 2020-12-20

**Authors:** 昆煜 张, 朝霞 戴, 思雅 刘, 丹 李, 达夫 杨, 赛琼 崔

**Affiliations:** 116021 大连，大连医科大学附属第二医院 The Second Affiliated Hospital of Dalian Medical University, Dalian 116021, China

**Keywords:** 肺肿瘤, 脑膜转移, 循环肿瘤DNA, 疗效评价, Lung neoplasms, Meningeal metastasis, Circulating tumor DNA, Response evaluation

## Abstract

**背景与目的:**

肺癌脑膜转移病死率极高。循环肿瘤DNA（circulating tumor DNA, ctDNA）已被证实含有肿瘤的基因组改变信息，并已被用于监测肿瘤的进展和对治疗的响应。对于存在脑膜转移瘤的患者，由于血脑屏障等因素的存在，外周血ctDNA不能反映脑部病灶的信息，此时脑脊液ctDNA作为检测样本能更好地体现颅内肿瘤的基因状态，指导临床对颅内病灶的靶向治疗。本研究旨在探究脑脊液ctNDA用于监测非小细胞肺癌（non-small cell lung cancer, NSCLC）脑膜转移的可行性以及脑脊液ctDNA检测对NSCLC脑膜转移的临床价值。

**方法:**

入组NSCLC脑膜转移患者21例，通过二代基因测序技术对患者的脑脊液及外周血样本进行基因检测，并进行脑脊液细胞学病理学检测和头颅核磁共振增强检查。

**结果:**

入组21例患者脑脊液中均检测到ctDNA。脑脊液ctDNA检测的灵敏性在脑膜转移诊断方面优于细胞学（*P* < 0.001）。脑脊液的基因突变检出率及基因突变丰度均高于血浆（*P* < 0.001）。脑脊液具有独特的基因谱。6例动态检测的患者中，脑脊液中ctDNA丰度变化均同时或早于临床疾病变化出现，可及时揭示耐药机制和监测复发趋势。

**结论:**

脑脊液ctDNA检出率高于细胞学及影像学；脑脊液ctDNA检测可展现脑膜转移病灶特有的突变图谱；脑脊液ctDNA动态监测对肺癌患者临床疗效具有提示意义。

## Abstract (en)

**Background and objective:**

The mortality rate of lung cancer meningeal metastasis is extremely high. Circulating tumor DNA (ctDNA) has been confirmed to be contain the genomic alterations present in tumors and has been used to monitor tumor progression and response to treatments. Due to the presence of blood-brain barrier and other factors, peripheral blood ctDNA cannot reflect the information of brain lesions for patients with meningeal metastases. However, cerebrospinal fluid ctDNA as a test sample can better reflect the genetic status of intracranial tumors and guide clinical targeted treatment of intracranial lesions. This study explored the feasibility of cerebrospinal fluid ctNDA for evaluating non-small cell lung cancer (NSCLC) meningeal metastasis and the potential clinical value of cerebrospinal fluid ctDNA detection in NSCLC meningeal metastasis.

**Methods:**

A total of 21 patients with NSCLC meningeal metastasis were included. Tumor genomic variation was performed on the cerebrospinal fluid and peripheral blood samples of patients by second-generation gene sequencing technology. The situation was examined, and pathological evaluation of cerebrospinal fluid cytology and head magnetic resonance imaging (MRI) enhanced examination were performed.

**Results:**

ctDNA was detected in the cerebrospinal fluid of 21 patients. The sensitivity of cerebrospinal fluid ctDNA detection was superior to cytology in the diagnosis of meningeal metastasis (*P* < 0.001). The detection rate and gene mutation abundance of cerebrospinal fluid were higher than plasma (*P* < 0.001). Cerebro-spinal fluid had a unique genetic profile. In 6 patients with dynamic detection, changes of ctDNA allele fraction occurred at the same time or earlier than clinical disease changes, which could timely monitor drug resistance mechanism and relapse trend.

**Conclusion:**

The detection rate of ctDNA in cerebrospinal fluid is higher than that in cytology and imaging. The detection of ctDNA in cerebrospinal fluid can reveal the specific mutation map of meningeal metastasis lesions. The dynamic monitoring of ctDNA in cerebrospinal fluid has hint significance for clinical response of lung cancer patients.

脑膜转移是恶性肿瘤细胞扩散至蛛网膜下腔进入脑脊液的结果^[1]^，约有3%-5%的晚期非小细胞肺癌（non-small cell lung cancer, NSCLC）患者会发生这种死亡率很高的并发症，伴有表皮生长因子受体（epidermal growth factor receptor, *EGFR*）驱动基因突变的NSCLC患者发生脑膜转移的几率更高，为9%-10%^[2, 3]^。临床上脑膜转移组织难以获取，因此我们对脑膜转移的发生发展机制了解有限，且因其具有高侵袭性及高发病率，脑膜转移仍为预后很差的肿瘤并发症^[4-6]^。液体活检的出现为患者提供了获取基因谱的新方法，改善了传统组织活检的诸多局限性，如无法克服肿瘤异质性、侵入性操作风险、样品制备误差等，在肿瘤诊断、筛查及预后等方面具有巨大潜力^[5, 7-9]^。美国食品药品监督管理局（Food and Drug Administration, FDA）于2016年首次批准液体活检作为肺癌伴随诊断手段，这是该领域的重要里程碑^[10]^。最常见的液体活检样本为外周血，研究显示循环肿瘤DNA（circulating tumor DNA, ctDNA）检测可用在NSCLC诊断预后等方面^[11-14]^，然而对发生脑转移的患者，血浆ctDNA检测效果不佳^[5, 15-17]^。例如，大多数具有中枢神经系统转移的NSCLC患者EGFR酪氨酸激酶抑制剂（EGFR-tyrosine kinase inhibitor, EGFR-TKI）失败后，在脑脊液中仍可检测到*EGFR*敏感突变，但在匹配血浆中的检测却少得多^[16]^。有研究^[17-19]^表明脑脊液作为ctDNA检测新媒介，能够更准确、更全面地反映脑部肿瘤基因突变情况。Ying等^[19]^研究显示脑转移患者脑脊液的ctDNA检出率优于外周血（81.5% *vs* 62.5%, *P*=0.008），且脑脊液ctDNA突变丰度液高于外周血（43.64% *vs* 4.58%）。吴一龙^[17]^、范云等^[18]^的研究中也观察到相同情况，脑脊液ctDNA可检测到更多的基因突变位点。

本研究入组21例NSCLC脑膜转移患者，对患者的脑脊液及外周血样本通过下一代测序技术进行基因检测，评估脑脊液ctDNA检测在脑膜转移分子诊断及动态监测患者临床疗效方面的能力。

## 1 材料与方法

### 1.1 材料

收集2018年3月1日-2020年1月31日于大连医科大学附属第二医院肿瘤内科就诊的NSCLC脑膜转移患者，分别收集每例患者的脑脊液及外周血样本，进行病理细胞学检查和下一代测序技术基因检测。同时完善患者临床资料，包括性别、年龄、用药方案、治疗情况等。

### 1.2 纳入标准及疗效评价标准

纳入标准即脑膜转移的诊断标准，包括：（1）脑脊液细胞学检查发现癌细胞，为脑膜转移诊断的金标准^[20]^。（2）对于脑脊液细胞学阴性的患者，需具备以下至少3条标准进行诊断：①其他原因无法解释的颅内压增高表现及脑膜刺激征，如头晕，头痛，定向障碍，呕吐，颈项强直，克氏征、布氏征、意识障碍，视力、听力、记忆力减退等神经功能症状^[20]^；②其他原因无法解释的脑脊液压力升高（> 180 mmH_2_O）；③典型的核磁共振成像（magnetic resonance imaging, MRI）等影像学表现（脑膜强化）；④脑脊液ctDNA阳性^[17]^（ctDNA中检测到肿瘤特有突变）。

对于影像学检查阴性的患者，根据以下标准进行疗效评价：①改善/稳定：临床症状改善或稳定，卡氏体能状态（Karnofsky performance status, KPS）评分不变或提升，脑脊液压力下降；②进展：临床症状加重，KPS评分减低，临床状况恶化，脑脊液压力上升^[21, 22]^。有影像学检查到可测量病灶的患者除以上标准外，使用实体瘤疗效评价标准（Response Evaluation Criteria in Solid Tumors, RESIST）1.1版结合脑转移瘤-神经肿瘤疗效评估（Response Assessment in Neuro-Oncology Brain Metastases, RANO-BM）标准进行疗效评价，对于MRI成像评估，蛛网膜下腔肿块被定义为可测量的病变。同时，除脑膜肿块以外的脑膜转移阳性表现（如线性或弥漫性增强）被定义为无法测量的病变。根据RESIST v1.1，所有无法测量的病变的消失都定义为完全缓解（complete response, CR），现有无法测量的病变的明确进展则定义为疾病进展（progressive disease, PD），而病变未见明显变化定义为疾病稳定（stable disease, SD）^[23]^。

临床症状评估根据RANO工作组提出的神经检查项目，主要对患者步态、肌力、感知、视觉、眼部运动、面部肌力、听觉、吞咽、意识、行为及患者治疗前出现的其他症状进行评价^[23, 24]^。

### 1.3 主要试剂

无酶水（Life technology, Cat: 10977-023）、无水乙醇（Sigma, Cat: E-7023, 500 mL）、Gentra Puregene Blood Kit Plus（1, 000 mL, QIAGEN, Cat: 158389）、QIAamp DNA FFPE Tissue提取试剂盒（QIAGEN, Cat: 56404）、QIAamp Circulating Nucleic Acid Kit（50 Rxn, QIAGEN, Cat: 551140）、KAPA HypePlus kit（KAPA, Cat: kk8504）、AxyPrep Fragment Select I beads（Corning, Cat: 14 223 162）、DNA capture probes（南京世和基因生物技术有限公司）xGen Blocking Oligos（1 nmol/μL in TE buffer），IDT Human Cot-1 DNA（Invitrogen, Cat: 15279-011）、Dynabeads M-270 Streptavidin（Life Technologies, Cat: 65305）、NEBnext Library Quant Kit for Illumina（500 rxn）（NEB, Cat: E7630L）xGen Lockdown Reagents（Hybridization and Wash Kit），IDT KAPA SYBR® FAST qPCR Kits（KAPA, Cat: KK4617）、HiSeq 3000/4000 SBS Kit（300 cycles, Illumina）、HiSeq 3000/4000 PE Cluster Kit（Illumina）。

### 1.4 实验步骤

#### 1.4.1 DNA提取和文库构建

将采集好的液体样本和相应的阴性对照样本在室温中进行预处理，离心得到上清液，置于预标记的冷冻管中放入-80 ℃冰箱保存。按试剂盒说明书步骤进行操作，进行样本DNA提取、磁珠分离纯化DNA，使用文库扩增试剂盒（KAPA Library Amplification Kit）进行DNA文库扩增，提纯DNA文库产物。

#### 1.4.2 探针富集

①DNA捕获探针与文库杂交，在基因组文库中加入封闭引物、杂交溶液、捕获探针以杂交过夜；②捕获文库产物的清洗和回收，使用Wash Buffer清洗捕获后的杂交产物，以降低非特异性杂交的背景；③捕获文库与链霉亲和素磁珠结合，将活化后的链霉亲和素磁珠与杂交产物混匀，孵育过夜；④磁珠捕获文库清洗，去除非特异结合的文库。

#### 1.4.3 上样测序

按照Hiseq 4000 User Guide准备测序试剂，将携有cluster的flow cell上机（Hiseq 4000, Illumina）。选用paired-end程序，进行双端测序，南京世和基因生物技术有限公司生产的425 panel作为肿瘤相关基因捕获探针。测序过程由Illumina提供的data collection software进行控制，并进行实时数据分析。

### 1.5 数据分析

使用人类基因组数据hg19作为参考分析对比检测到的数据，进一步筛选分析单核苷酸变异和拷贝数变异类型，过滤错配及低质量序列。根据千人基因组数据库、dbSNP数据库等进一步对比结果，筛选肿瘤特有突变。统计学分析均使用IBM SPSS Statistics 21完成，*P* < 0.05为差异有统计学意义。使用Graphpad Prism 8.0及R语言3.6软件完成绘图。

## 2 结果

### 2.1 研究对象一般情况描述

本研究共入组NSCLC脑膜转移患者21例，男性5例，女性16例，年龄39岁-78岁，中位年龄56岁。21例患者均采集脑脊液ctDNA进行基因检测，同时进行脑脊液细胞学病理学检测和头颅MRI检查，其中，17例（17/21）患者脑脊液细胞学阳性，3例患者MRI阳性。13例（13/21）患者在检测前有EGFR-TKIs治疗史；入组的21例脑膜转移患者中，其中仅患者P20脑脊液中检测到*EGFR* T790M突变，但由于奥西替尼可以作为*EGFR*敏感突变的一线用药，且是脑转移患者的首选用药，13例患者确诊为脑膜转移后均使用奥西替尼治疗（表 1）。

**表 1:** 患者一般状况 Clinical characteristics of patients

Patient	Age (yr)	Gender	Stage	Cytology	MRI	Treatment before LM	PFS (mon)	EGFR p.T790M	TKIs after LM	PFS（mon）
P1	65	Female	Ⅰ	+	-	Chemo+bevacizumab	6	-	Osimertinib	19 (lost)
P2	63	Female	Ⅳ	+	+	Chemo+bevacizumab	14	-	Osimertinib	17
P3	56	Female	Ⅳ	+	+	Gefitinib	8	-	Osimertinib	7
P4	53	Female	Ⅳ	+	-	Gefitinib	6	-	Osimertinib	11
P5	49	Male	Ⅳ	+	-	Icotinib	6	-	Osimertinib	1
P6	63	Male	Ⅳ	-	-	Chemo+bevacizumab	-	-	-	-
P7	54	Female	Ⅳ	+	-	Apatintib	21	-	Osimertinib	3 (lost)
P8	59	Female	Ⅳ	+	-	Gefitinib	15	-	Osimertinib	7
P9	67	Female	Ⅳ	+	-	Icotinib	12	-	-	（lost）
P10	43	Female	Ⅱ	+	-	Gefitinib	14	-	Osimertinib	3
P11	60	Female	Ⅳ	+	-	Icotinib	12	-	-	4
P12	48	Female	Ⅳ	+	-	Gefitinib	0.67	-	Osimertinib	（lost）
P13	57	Female	Ⅳ	+	-	Icotinib	7	-	Osimertinib	3
P14	39	Female	Ⅳ	+	-	Chemo+bevacizumab	-	-	-	-
P15	74	Female	-	+	-	-	-	-	Osimertinib	-
P16	50	Male	Ⅳ	-	-	Gefitinib	5	-	-	-
P17	48	Female	Ⅱ	-	-	Icotinib	-	-	-	-
P18	78	Male	Ⅳ	-	-	Gefitinib	30	-	-	-
P19	43	Male	Ⅱ	+	-	Icotinib	20	-	Osimertinib	8
P20	62	Female	Ⅳ	+	+	Surgery	-	+	Osimertinib	2 (lost)
P21	50	Female	-	+	-	Chemo	-	-	-	-
MRI: magnetic resonance imaging; LM: leptomeningeal metastases; PFS: progression-free survival; EGFR: epidermal growth factor receptor; TKI: tyrosine kinase inhibitor; Chemo: chemotherapy; lost：lost to follow up.										

21例患者均进行脑脊液和血浆样本的采集，5例患者有既往肺组织FFPE标本送检。肺组织中检测率最高的为*EGFR*（80%）及*TP53*（60%）驱动基因突变，还检测到*ALK*、*RB1*、*TRET*等肿瘤特有突变（图 1）。此外，6例患者多次取脑脊液及血浆样本进行基因检测，评估脑脊液检测结果与疗效的相关性。

**图 1:**
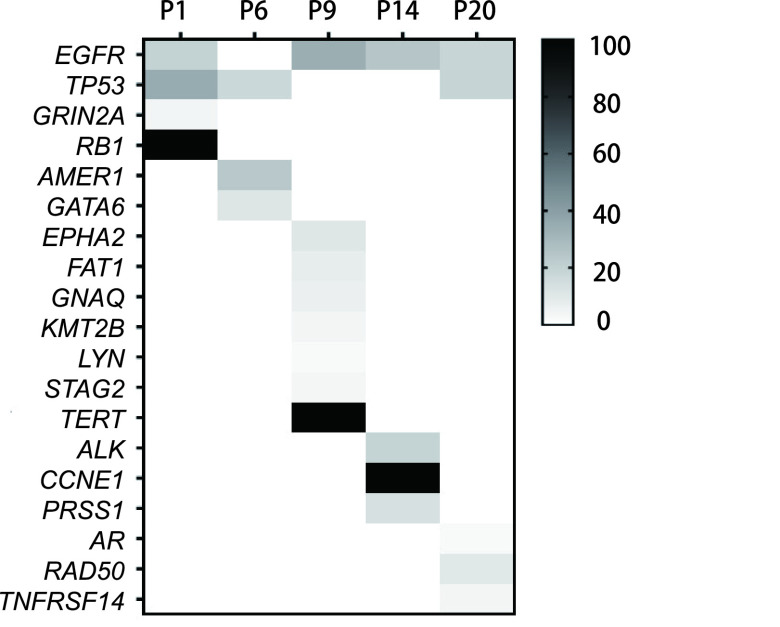
患者肺组织基因突变谱。5例患者（P1、P6、P9、P14、P20）既往有肺组织留存，进行基因检测，检测率最高为*EGFR*（80%）及*TP53*（60%）突变，还可检测到*ALK*、*RB1*等基因突变。 Gene mutation spectrum of patients' lung tissues. Five patients (P1, P6, P9, P14, P20) had performed genetic testing of lung tissue. The highest detection frequencies were *EGFR* (80%) and *TP53* (60%) mutations. *ALK*, *RB1* and other gene mutations can also be detected.

### 2.2 脑脊液基因检测结果一般描述

本研究分别对21例NSCLC患者匹配的脑脊液和外周血进行下一代测序技术检测，脑脊液ctDNA共检测到63种基因突变，共114个突变位点。在这些突变结果中，突变频率最高是*EGFR*（86%）和*TP53*（81%），其他驱动基因突变还包括*ALK*、*ERBB2*、*PIK3CA*、*MET*、*NXK2-1*和*NTRK1*扩增等。

*EGFR*突变亚型中发生率最高的是外显子19的非移码缺失突变（38%）和外显子21的L858R错义突变（38%），1例患者（P20）同时伴随*EGFR* T790M突变，1例患者（P8）同时伴有*EGFR* L718Q突变，1例患者为*EGFR* KDD罕见突变，另外1例患者脑脊液中检测到了L861Q罕见突变。此外，脑脊液中还检测到了新的驱动基因突变：2例*ALK*基因变异，2例*ERBB2*扩增，1例*PIK3CA*基因突变，1例*MET*基因突变。

### 2.3 脑脊液ctDNA具有特有的突变图谱

检测过程中，针对脑脊液及血浆ctDNA检测匹配相应的白细胞阴性对照，同时结合检测公司的生信算法优化和数据库积累，更好地排除了克隆性造血的干扰。脑脊液检测到50个独有的基因突变和拷贝数变化，包括*SMAD4*、*PIK3C3*、*CTNNB1*、*MET*等基因突变和*EGFR*、*HER-2*、*MYC*、*NKX2-1*扩增及*RB1*拷贝数缺失等。

5例患者有既往肺部组织样本留存，也对其进行送检及结果分析（图 1）。患者脑脊液中的驱动基因突变如*EGFR*、*ALK*突变与组织中一致，仅患者P1脑脊液中出现*TP53*拷贝数缺失，患者P20脑脊液中出现*EGFR* T790M突变，组织及血浆中均中未检测到，为脑脊液独有。

### 2.4 脑脊液ctDNA检出率高于细胞学及影像学

本研究中21例患者脑脊液ctDNA阳性率为100%（21/21），细胞学检查阳性率为81%（17/21）。对6例动态监测的患者共进行了21次脑脊液基因及细胞学检查，脑脊液*EGFR*驱动基因阳性检测率为100%（21/21），而细胞学阳性率仅为57%（12/21）。患者P13首次脑脊液检测即检测到肿瘤特有基因突变，脑脊液细胞学检测为阴性，直至3个月后脑脊液细胞学才提示患者脑转移。

影像学的评估中，仅有3例患者有MRI阳性表现，其中1例（1/3）患者（P2）在确诊为脑膜转移18个月后复查MRI首次得到阳性结果，MRI灵敏度仅有16%。

### 2.5 脑脊液ctDNA优于外周血

本研究对21例匹配的的脑脊液及血浆样本进行基因检测，脑脊液的基因检出率为100%（21/21），而血浆的检测率检出率仅为52%（11/21），脑脊液检测率显著高于血浆（*P* < 0.001）。脑脊液中共检测出63种基因突变，血浆中仅检测出15种基因突变。脑脊液ctDNA基因突变丰度显著高于外周血样本（中位数：22.79% *vs* 0.57%，*P* < 0.001）（图 2）。

**图 2:**
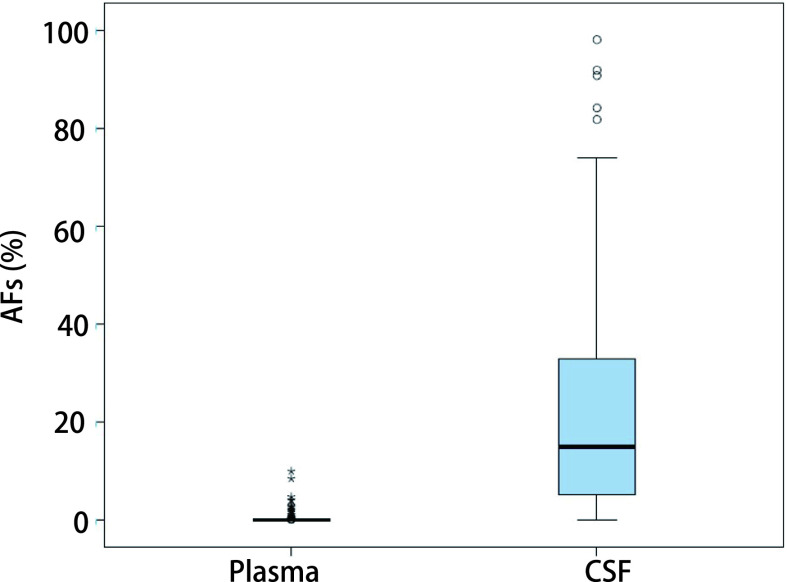
脑脊液与血浆基因突变丰度对比。对21例匹配的的脑脊液及血浆样本进行基因检测，脑脊液检测率大大高于血浆（*P* < 0.001）。突变丰度：在该位点所有的等位基因中，突变的等位基因的占比（相对野生型等位基因）。 Comparison of allele fraction between cerebrospinal fluid and plasma. Genetic testing of 21 matched cerebrospinal fluid and plasma samples, the detection rate of cerebrospinal fluid is much higher than that of plasma (*P* < 0.001). CSF: cerebrospinal fluid. Mutation abundance: the proportion of mutant alleles (relative to wild-type alleles) among all alleles at this locus. AF: alleles frequency.

### 2.6 脑脊液ctDNA监测对于患者临床治疗的提示意义

本研究中对6例NSCLC患者进行脑脊液及外周血的动态监测。6例患者在确诊为脑膜转移后均开始服用奥西替尼进行治疗。患者P1治疗过程中一直保持病情稳定，脑脊液中*EGFR*的突变丰度由42.6%下降至22.1%，后又上升至43.7%，波动不大，维持在40%左右（图 3）。患者P4及P13的脑脊液*EGFR*基因突变丰度均在上升后落回原来的水平，虽然有波动但并无明显的上升趋势（图 4、图 5），监测期间患者病情保持稳定，与P1的脑脊液监测结果相同，均与临床表现一致。

**图 3:**
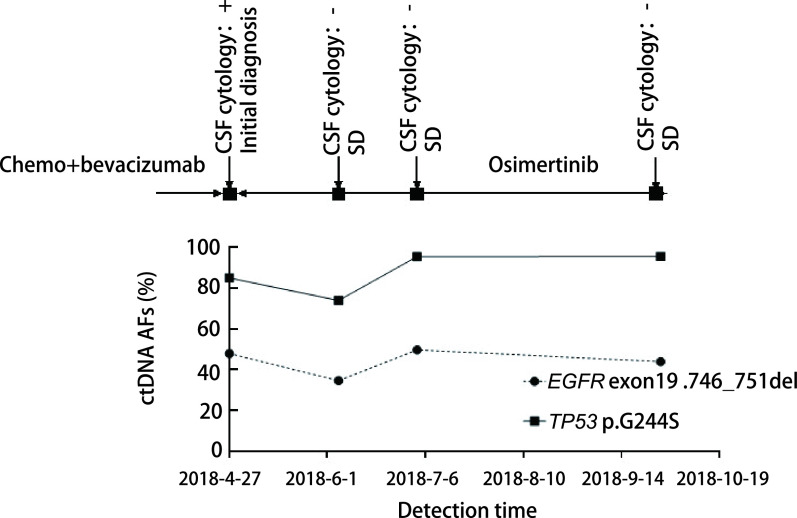
患者P1动态监测丰度结果。患者P1治疗过程中一直保持病情稳定，脑脊液中*EGFR*及*TP53*的突变丰度未出现显著的波动。 Dynamic monitoring results of patient P1. The patient's condition remained stable during the treatment of P1, and the mutation abundance of *EGFR* and *TP53* in cerebrospinal fluid did not fluctuate much. ctDNA: circulating tumor DNA; SD: stable disease.

**图 4:**
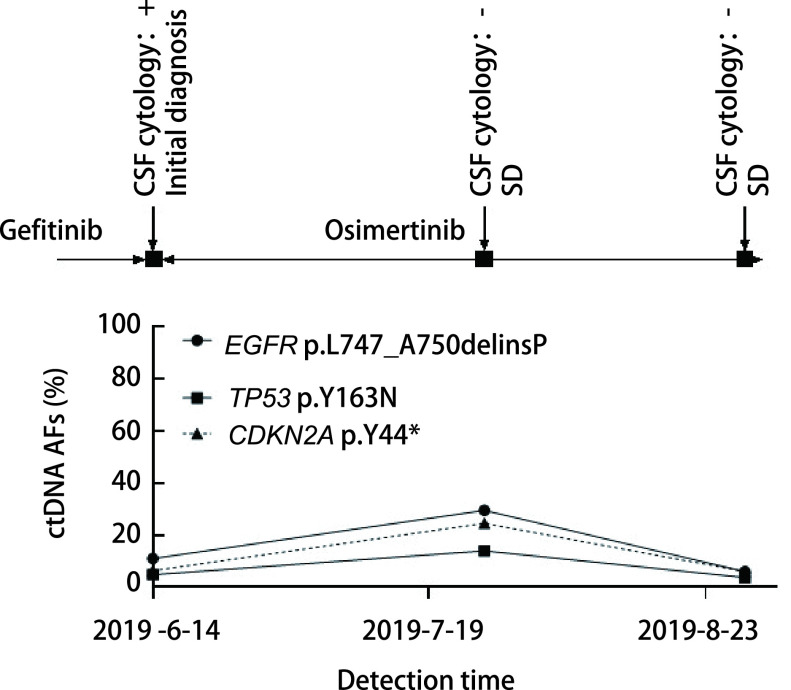
患者P4动态监测丰度结果。患者P4 ctDNA丰度保持稳定，脑脊液*EGFR*基因突变丰度均在上升后落回原来的水平，虽然有波动但并无明显的上升趋势。 Dynamic monitoring results of patient P4. The abundance of ctDNA in P4 remained stable, and the abundance of cerebrospinal fluid *EGFR* gene mutations fell back to the original level after rising. Although there was fluctuation, there was no obvious upward trend.

**图 5:**
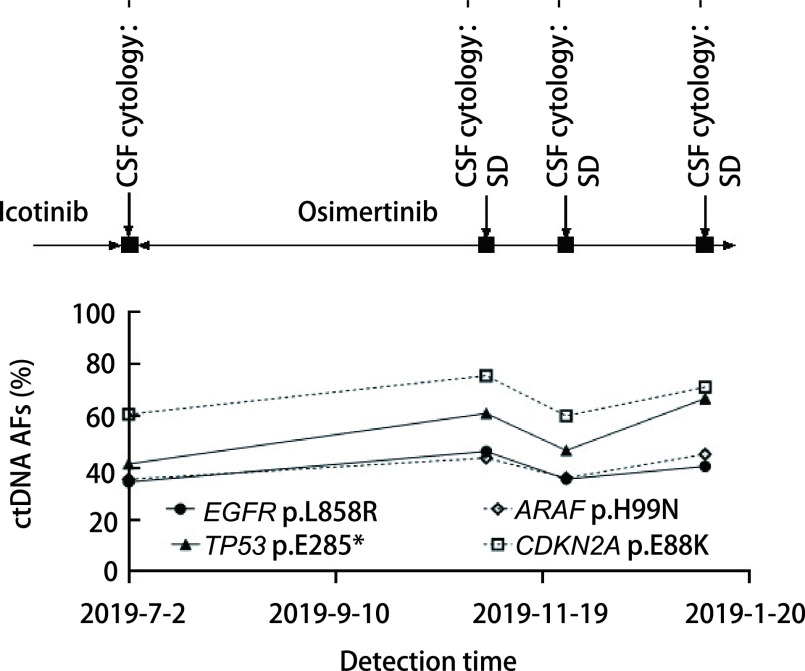
患者P13动态监测丰度结果。患者P13 ctDNA一直保持稳定，脑脊液*EGFR*基因突变丰度均在上升后落回原来的水平并再次上升，虽然有波动但并无明显的上升趋势。 Dynamic monitoring results of patient P13. The ctDNA of P13 has remained stable, and the abundance of cerebrospinal fluid *EGFR* gene mutations have fallen back to their original levels and increased again after rising. Although there is fluctuation, there is no obvious upward trend.

此外本研究中还可观察到部分脑脊液ctDNA检测结果先于临床表现反映患者病情变化的现象。患者P2使用奥西替尼后病情得到有效控制，其脑脊液由首次检测突变丰度42.6%，11个月后降至22.1%；而在第4次复查时，患者临床症状加重，MRI检查首次检测到脑膜转移强化灶，病情进展，脑脊液*EGFR*基因突变丰度上升至43.7%。值得注意的是，患者在第3次复查时检测到了新发*PIK3R1*突变，但当时临床未评价癌症进展（图 6）。

**图 6:**
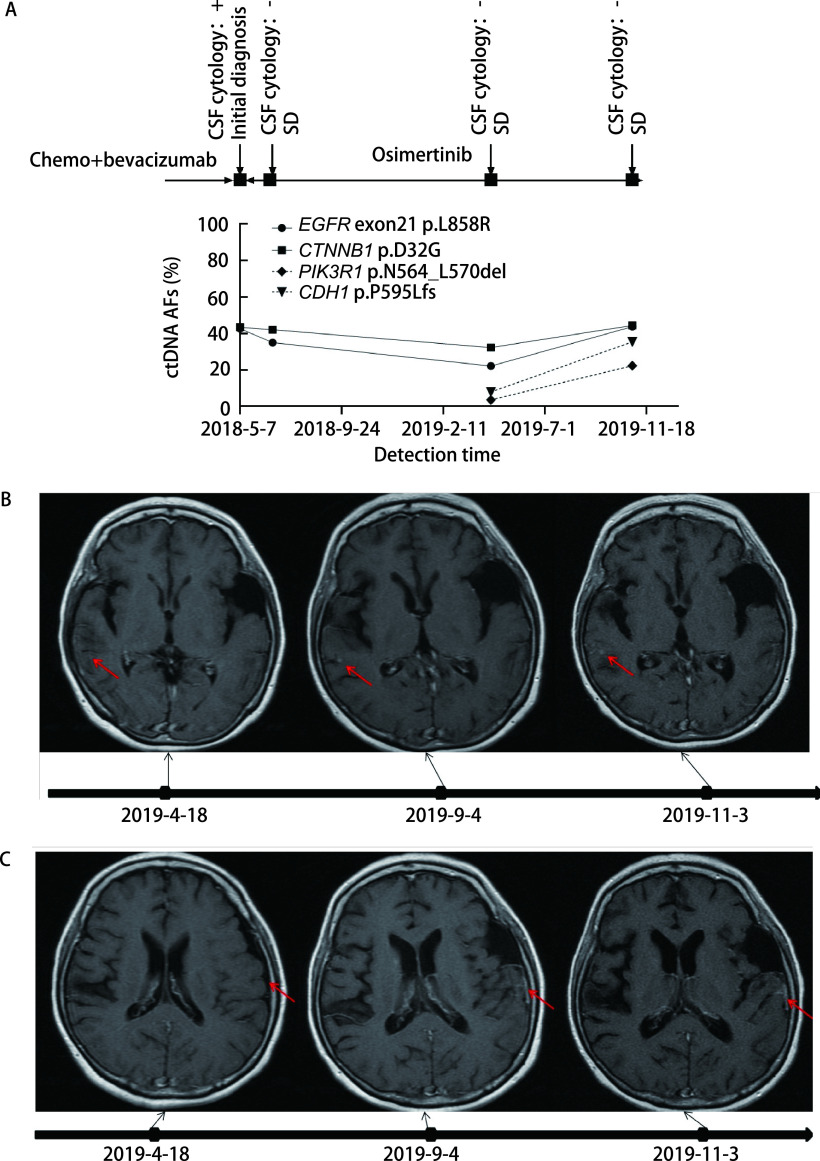
患者P2动态监测丰度结果。A：患者P2使用奥西替尼后病情得到有效控制，其脑脊液由首次检测突变丰度下降；而在第4次复查时，患者临床症状加重，脑脊液*EGFR*突变丰度上升。B：患者P2 MRI图像：2019-4-18箭头指示局部未见异常强化；2019-9-4出现结节样脑膜强化；2019-11-3显示强化范围扩大。C：患者P2 MRI图像：2019-4-18箭头指示局部未见异常强化；2019-9-4出现片状脑膜强化；2019-11-3显示脑膜强化较前相仿。 Dynamic monitoring results of patient P2. A: The patient's condition was effectively controlled after taking Osimertinib in P2, and the abundance of his cerebrospinal fluid decreased from the first detection of the mutation; while at the fourth review, the patient's clinical symptoms worsened and the abundance of cerebrospinal fluid *EGFR* mutation increased. B: MRI image of patient P2: The arrow indicates that there is no abnormal enhancement in the area on April 18, 2019; Nodular meningeal enhancement appears on September 4, 2019; On November 3, 2019, the scope of enhancement is enlarged. C: MRI image of patient P2: The arrow indicates that there is no abnormal enhancement in the area on April 18, 2019; Sheet-like meningeal enhancement appears on September 4, 2019. On November 3, 2019, the meningeal enhancement is similar to before.

患者P3在第3次复查时脑脊液*EGFR*基因突变丰度上升至37.2%，而当时的疗效评价为疾病稳定（stable disease, SD），未体现出疾病进展征象；3个月后患者再次进行复查，检查结果提示病情进展，而脑脊液基因突变丰度与第3次结果相比并无明显变化（图 7）。

**图 7:**
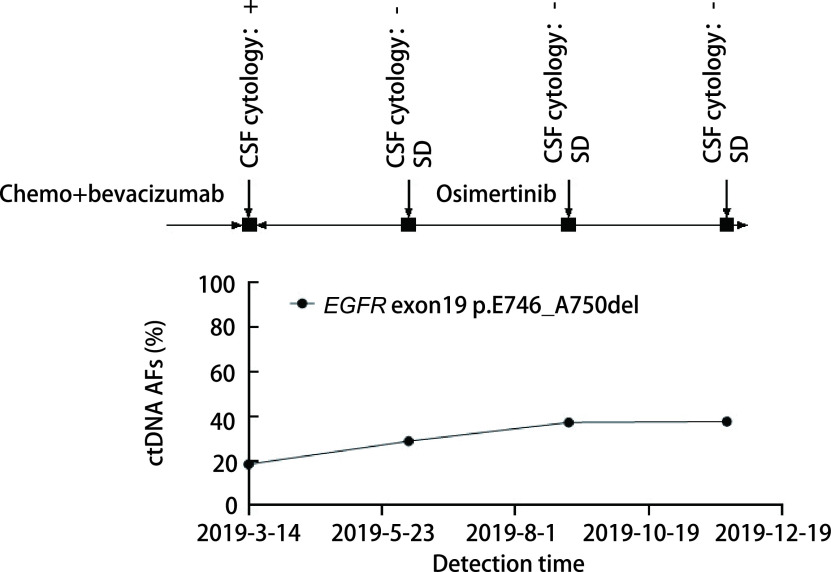
患者P3动态监测丰度结果。患者P3在第3次复查时脑脊液*EGFR*基因突变丰度升高，3个月后患者再次进行复查，脑脊液基因突变丰度与第3次结果相比并无明显变化。 Dynamic monitoring results of patient P3. The P3 patient's cerebrospinal fluid *EGFR* gene mutation abundance increased at the third review, and the patient was reexamined three months later. The cerebrospinal fluid gene mutation abundance did not change significantly from the third result.

患者P20在确诊1个月后复查，脑脊液ctDNA丰度上升，同时患者脑脊液中检测到多种新增基因拷贝数变异，包括*MYC*基因扩增、*FGFR4*基因扩增等，但患者病情稳定，无进展征象（图 8）。

**图 8:**
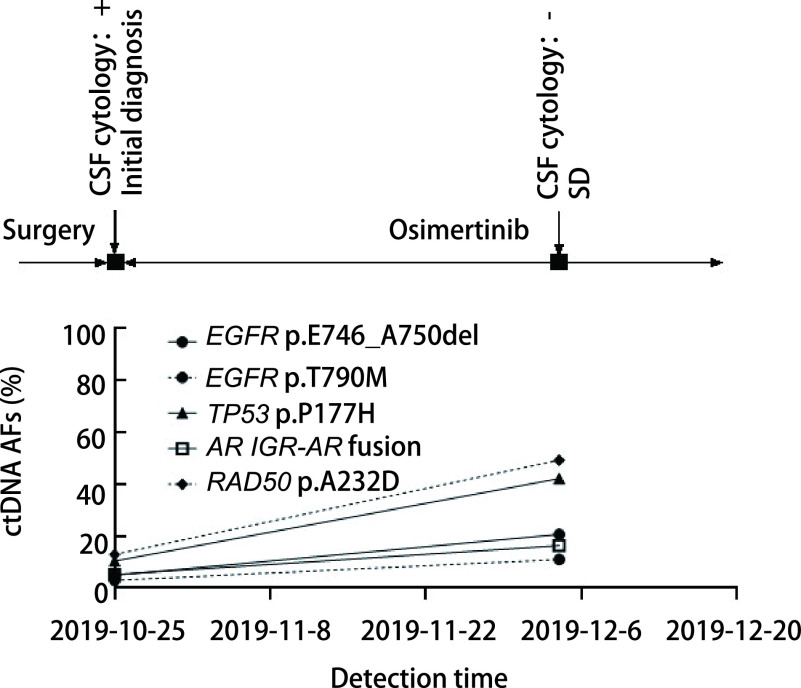
患者P20动态监测丰度结果。患者P20在确诊1个月后复查，脑脊液ctDNA丰度上升。 Dynamic monitoring results of patient P20. P20 was reexamined one month after diagnosis, and the abundance of ctDNA in cerebrospinal fluid increased.

## 3 讨论

脑膜转移肿瘤与脑脊液直接接触，释放的ctDNA片段可直接进入脑脊液，而由于血脑屏障的存在，ctDNA难以进入血液；且外周血中存在大量正常组织来源的游离DNA，ctDNA占比较小，检测很容易受干扰。多项研究^[16-19]^表明脑脊液在反映脑膜转移灶基因突变情况方面显著优于外周血，本研究结果显示脑脊液基因检出率及丰度均明显高于血浆，因此尽管腰椎穿刺的侵入性稍大，应用脑脊液ctDNA检测了解颅内病灶情况仍十分必要。

本研究中检测到多种脑脊液中独有的基因，这些基因可能与脑膜转移灶的发生及发展相关，可帮助肿瘤细胞突破血脑屏障迁移至颅内，并适应中枢神经系统特殊的微环境。脑脊液检查结果中大部分特有的基因突变如*MYC*基因扩增、*CTNNB1*、*SMAD4*基因突变等，均与脑膜转移机制相关^[25-28]^。Klotz等^[25]^的研究中发现，*MYC*基因过表达可能是肿瘤细胞适应颅内微环境的机制之一，能够减轻颅内氧化应激状态帮助肿瘤细胞生存。我们的研究中，脑脊液特有的基因突变中还可观察到多种与PI3K/AKT/mTOR通路相关的基因，如*FGFR3*、*FGFR4*、*PIK3R1*、*AKT3*、*STK11*等^[29]^。PI3K/AKT/mTOR通路是一种在多种肿瘤中激活的细胞内信号通路，其激活参与了肿瘤的进展和耐药^[30, 31]^，同时是EGFR及ALK靶向药物的重要耐药机制之一^[32, 33]^。一项关于肺鳞癌患者的研究^[34]^发现伴有*PI3K*相关基因突变的患者疾病进展快，预后较差且颅内转移发生率较高，表明PI3K/AKT/mTOR通路的激活可能与脑膜转移的发生有关。范云等^[18]^研究发现脑膜转移患者中*PIK3CA*基因突变检测率较一般肺腺癌人群高，同样表明PI3K通路可能参与脑膜转移。

脑脊液检测到了大量基因的拷贝数变异，表明拷贝数变异与脑膜转移之间有密切联系。拷贝数变异的水平与较短的无进展生存期相关^[35, 36]^，说明脑膜转移灶拷贝数变异是其预后不良的重要机制之一。*TP53*和*RB1*拷贝数缺失，这两种基因突变与小细胞肺癌转化密切相关，而这种组织学表型转化是NSCLC对靶向药物耐药的机制之一^[35]^。14%（3/21）的入组患者中检测到*NXK2-1*扩增，*NKX2-1*是一种转录因子，研究^[37]^表明其突变通过上调或激活EGFR的下游通路影响NSCLC细胞增殖，且敲低*NXK2-1*能增强EGFR抑制剂的作用。在吴一龙等^[17]^、范云等^[18]^的研究中均同样报道了脑脊液中检测到大量独有的基因突变及拷贝数变异，且大部分与DNA损伤修复及细胞周期调节等通路相关。由此可见，通过对脑脊液特有的基因谱进行分析，可以深入了解脑膜癌转移及进展的机制，进而预防其发生。

目前脑膜转移诊断的金标准为脑脊液细胞学检查，但这种检验手段的阳性率不足60%^[38, 39]^。由于ctDNA灵敏度更高，在诊断方面表现出比脑脊液细胞学更大的潜力。有研究^[40]^通过脑脊液ctDNA检测到了脑脊液细胞学结果阴性患者的脑膜转移灶。我们的研究结果提示：即使是确诊患者，多次检测得到的脑脊液细胞学检出率仍远低于ctDNA检出率（*P* < 0.001）。患者P13首次检测仅脑脊液ctDNA显示阳性，复查时才获得细胞学阳性结果。相比较脑脊液细胞学检测，MRI检测结果阳性率更低，仅为16%。患者P2在确诊18个月后病情进展时MRI才观察到脑膜转移征象，且这例患者同期进行的脑脊液细胞学检查结果为阴性，说明即使在脑膜转移灶进展阶段，脑脊液细胞学灵敏度仍较低。Zhao等^[21]^对脑膜肿瘤患者进行脑脊液ctDNA、细胞学及影像学检查，同样发现ctDNA的灵敏度（100%）远高于细胞学（71.43%）和影像学（62.86%）。这些结果表明，脑脊液ctDNA检测灵敏度优于细胞学及影像学，可以作为脑脊液细胞学及MRI检测阴性结果的补充诊断方法，同时，脑脊液检测优于细胞学检测和MRI的是可通过脑脊液检测结果辅助制定患者的治疗方案。

脑脊液ctDNA检测也可用于指导NSCLC脑膜转移患者临床治疗的药物选择。尽管入组患者大部分都有一代EGFR-TKIs治疗史，但21例患者中仅1例患者在脑脊液中检测到T790M突变，突变率很低。有研究^[41-43]^报道了类似的现象，在吉非替尼耐药的患者脑脊液中仅检测到14.3%的T790M突变，远低于既往报道的颅外病灶T790M突变率（约50%）。这可能是因为血脑屏障的存在导致颅内血药浓度低，未达到刺激肿瘤细胞耐药水平，而脑脊液中检出大量高丰度的*EGFR*靶向药物敏感突变也提示颅内药物未达到最佳治疗水平^[41, 42]^。Huang等^[43]^进行的一项回顾性研究报道验证了这种猜想，2例患者在接受埃克替尼治疗后病情进展，但在脑脊液中未检测到T790M突变，仍存在L858R突变，据此予患者药物加量治疗而未换药后，这2例患者分别保持SD 4个月和12个月，证明其治疗有效。本研究中还发现了其他罕见突变如*EGFR* L816Q突变及*EGFR* KDD突变。*EGFR* KDD即*EGFR*激酶结构域重复，对EGFR-TKIs尤其是第二代药物阿法替尼敏感^[44]^。患者P8脑脊液中还检测到了*EGFR* L718Q突变，它是继*EGFR* T790M突变后的三级突变^[45]^。这例患者在脑脊液检测前有奥西替尼用药史，可推测脑脊液中*EGFR* L718Q突变是其出现耐药的表现，提示脑脊液基因检测结果可提示患者耐药机制，以做出相应药物调整，延长患者生存期。

ctDNA可以来源于肿瘤的任意部位，能克服肿瘤的时间及空间异质性，成为基因检测的优秀样本来源，且侵入性小^[8]^，具有成为动态监测手段的优势。RANO关于脑膜转移疗效评价的报道中提到，可以应用脑脊液细胞学结果来评估脑膜转移患者治疗疗效，但Xu等^[23]^在研究中发现，即使是有着最严格治疗方案的患者也很难得到脑脊液细胞学阴性结果，这说明脑脊液细胞学作为疗效评价手段不够准确，在临床中并不适用。本研究对多例患者进行了多次脑脊液ctDNA检测，通过分析患者脑脊液基因突变丰度和临床疾病状态评价可以看出，脑脊液中基因突变丰度尤其是驱动基因突变丰度的变化基本与患者的疾病评估结果吻合度高。值得注意的是，个别患者在脑脊液基因丰度升高或出现新耐药突变基因时并未体现出病情进展，而是在下一次复查时临床评价为疾病进展，这可以说明脑脊液不仅能反映患者疾病状态，还能在病情发展前期未出现临床症状时做出预警。本研究中观测到的脑脊液细胞学结果与患者病情并无明显联系，可见脑脊液ctDNA能更准确地反映患者病情变化，可根据丰度变化判断患者病情进展情况，同时根据是否有新基因出现可监测脑膜转移肿瘤细胞演变情况，及时调整治疗策略。

本研究具有一定的局限性。首先，本研究样本量较小，检验效能较低；其次，由于是回顾性研究，患者的临床资料收集不够全面，且不能根据基因检测结果对患者的治疗方案进行干预。

综上所述，脑脊液ctDNA检测可展现脑膜转移病灶特有的突变图谱，可作为NSCLC脑膜转移的补充诊断手段，其动态监测对肺癌患者临床疗效具有提示意义。
